# Conserved aging-related signatures of senescence and inflammation in different tissues and species

**DOI:** 10.18632/aging.102345

**Published:** 2019-10-12

**Authors:** Emanuel Barth, Akash Srivastava, Milan Stojiljkovic, Christiane Frahm, Hubertus Axer, Otto W. Witte, Manja Marz

**Affiliations:** 1 Bioinformatics/High Throughput Analysis, Faculty of Mathematics and Computer Science, Friedrich Schiller University Jena, Jena, Germany; 2 FLI Leibniz Institute for Age Research, Jena, Germany; 3 Hans Berger Department of Neurology, Jena University Hospital, Jena, Germany; 4 European Virus Bioinformatics Center (EVBC), Jena, Germany

**Keywords:** aging, inflammaging, senescence, RNA-Seq, transcriptomics

## Abstract

Increasing evidence indicates that chronic inflammation and senescence are the cause of many severe age-related diseases, with both biological processes highly upregulated during aging. However, until now, it has remained unknown whether specific inflammation- or senescence-related genes exist that are common between different species or tissues. These potential markers of aging could help to identify possible targets for therapeutic interventions of aging-associated afflictions and might also deepen our understanding of the principal mechanisms of aging. With the objective of identifying such signatures of aging and tissue-specific aging markers, we analyzed a multitude of cross-sectional RNA-Seq data from four evolutionarily distinct species (human, mouse and two fish) and four different tissues (blood, brain, liver and skin). In at least three different species and three different tissues, we identified several genes that displayed similar expression patterns that might serve as potential aging markers. Additionally, we show that genes involved in aging-related processes tend to be tighter controlled in long-lived than in average-lived individuals. These observations hint at a general genetic level that affect an individual’s life span. Altogether, this descriptive study contributes to a better understanding of common aging signatures as well as tissue-specific aging patterns and supplies the basis for further investigative age-related studies.

## INTRODUCTION

The phenomenon of biological aging is still far from satisfactorily explained due to its highly complex nature. Aging is often described as a decline in cellular functions over time and the cause of several severe diseases, such as cardiovascular diseases, neurodegenerative diseases or cancer [1]. Because aging is incompletely understood, a decades-old and still ongoing debate exists on the true source of aging, giving rise to a variety of competing theories. Senescence and inflammatory processes are two of the most common themes within these discussions of the molecular driving forces of aging. Cellular senescence is a state in which permanent replication is halted, and thus cells are unable to further proliferate, and their overall function is strongly diminished [2]. Senescence is meant to be a protective mechanism, stopping further proliferation if cells are on the verge to turn into malignant tumor cell due to severe DNA damage, for example because of telomere shortening. During an organism’s lifetime, most cells continuously undergo proliferation and cell division and reach the state of senescence at their own pace, when their telomeres have reached a certain shortening threshold. Over time, this process leads to an accumulation of senescent cells accompanied by loss of function and integrity of the respective tissues, which reflects the close connection of senescence with aging [3]. Recently, cellular senescence was described as the “nexus of aging” by Bhatia-Dey et al., suggesting it as the main driver of the aging process [4].

Another systemic process, that is observed in most tissues with age is the increased release of proinflammatory messenger substances. As a consequence, low-grade chronic inflammatory processes slowly but irresistibly begin to damage organs and are viewed as the cause of other age-related chronic diseases, such as Alzheimer’s disease, osteoporosis or diabetes [5, 6]. This state of chronic age-dependent inflammation is also suspected as one of the main causes of biological aging and was described as “inflammaging” [7]. Miquel et al. proposed an integrative oxidation-inflammation theory of aging, arguing that chronic oxidative stress originating from mitochondria leads to senescence in cells of the regulatory systems, such as the immune system [8]. Unarguably, senescence and inflammation processes are strongly connected and contribute to an organism’s aging phenotype as well as its rate of aging. Therefore, understanding how those systemic processes are regulated during aging in different species and tissues could supply many answers related to biological aging itself.

Here we present a descriptive transcriptomic study on the age-dependent genetic changes of senescence and inflammation in the four evolutionarily distinct species of *Homo sapiens*, *Mus musculus*, *Danio rerio* and the short-lived fish *Nothobranchius furzeri* and up to four different tissues (brain, blood, liver, and skin). Our aim was to identify potential markers of aging across species and tissues by comparing a young mature time point against an aged and old-aged time point. We report 26 different genes that showed consistent upregulation or downregulation towards old age in multiple tissues and discuss their roles in aging. Furthermore, we identified several genes that were similarly regulated during aging among the investigated species in a tissue-specific manner. Additionally, we observed a stricter control of gene expression of aging-related processes in the rather old-aged individuals compared with the aged individuals. We conclude that certain of the identified genes that show a conserved age-dependent expression pattern are potentially interesting targets for therapeutic developments designed to achieve healthier aging.

## RESULTS AND DISCUSSION

### Gene expression discriminates among tissues more strongly than ages

**Figure 1:**
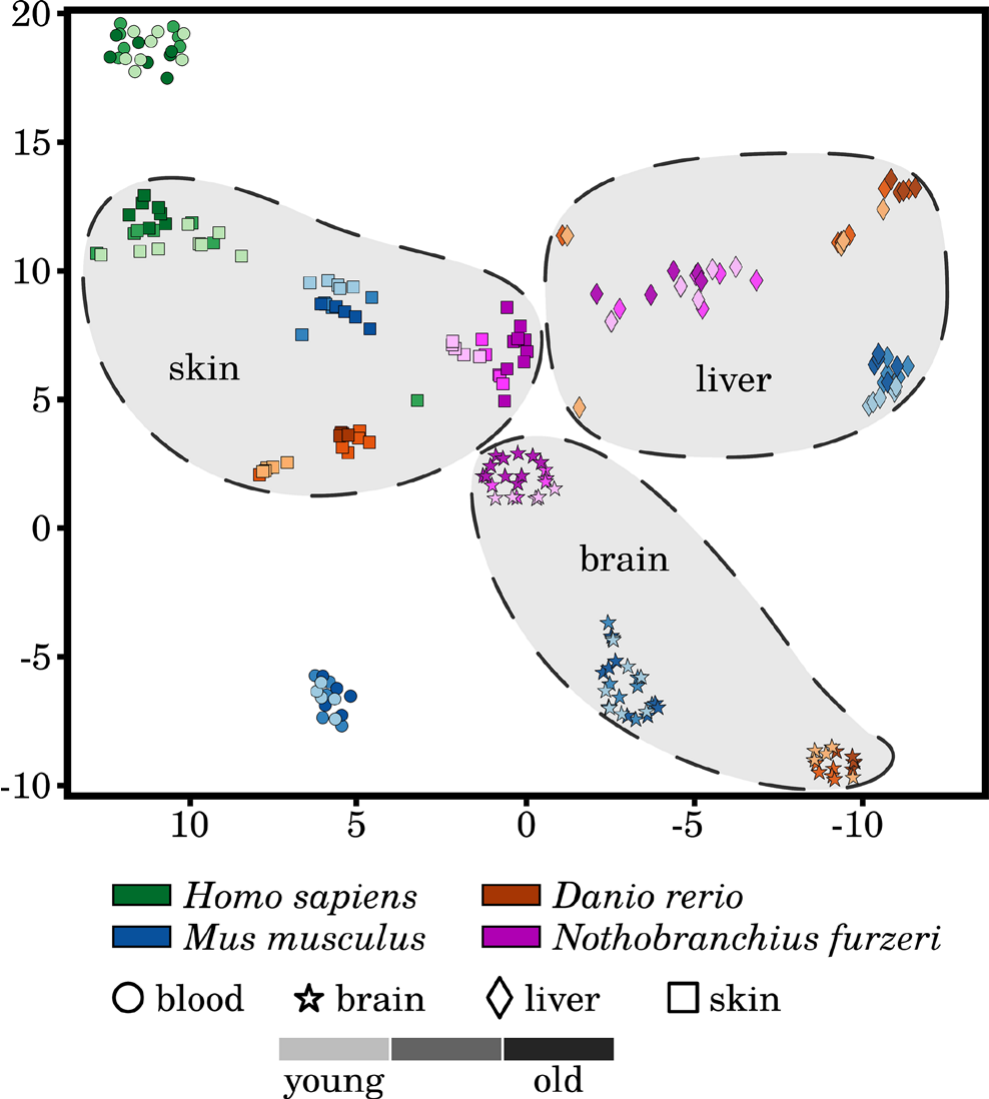
**A t-distributed stochastic neighbor embedding (t-SNE) of the analyzed RNA-Seq libraries.** All RNA-Seq samples were clustered based on the expression patterns of the selected senescence and inflammation related genes, utilizing the t-SNE approach. All tissues form distinctive species-specific and non-overlapping clusters with exception to very few single outliers. Additionally, larger species-independent tissue clusters were drawn to improve the visualization of the data. However, the three different time points did not generally separate in independent clusters of their own. A weak segregation can only be observed among the mature and old-aged skin samples of all four species. For more information, see Supplementary Data 10.

To evaluate the homogeneity of the investigated transcriptomic data, we performed t-SNE dimensionality reduction of the RNA-Seq libraries based on the measured expression strengths of our 464 preselected senescence- and inflammation-associated genes (see Figure 1). We observed a homogeneous clustering of the samples with respect to species and tissue, with almost no segregation of the three different ages within these clusters. This observation was already made and reported in similar studies based on different sets of genes and indicates that aging is a relative subtle process, at least on the transcriptional level [9]. Nevertheless, separations between the youngest and oldest time point can be observed within the skin clusters of all four species but to a much weaker extent in the human samples. This observation might suggest a pronounced and conserved difference in the activity of senescence and inflammation processes during skin aging of evolutionarily distinct species. This observation and the fact that the skin is also relatively easily accessible makes it especially interesting for interspecies comparisons of age-related senescence and inflammation. However, it remains to be further investigated whether this observation can be made in similar transcriptomic studies.

Another observation is that although the different tissue samples form species-specific clusters, they are still well separated from each other, *i.e.*, forming tissue-specific clusters, with the exception to the *Homo sapiens* and *Mus musculus* blood samples. This observation hints that the similarities of the inflammaging process among the same tissues of different species prevails over any systematic effect within one individual.

### Expression variance is more controlled in long-lived individuals

**Figure 2:**
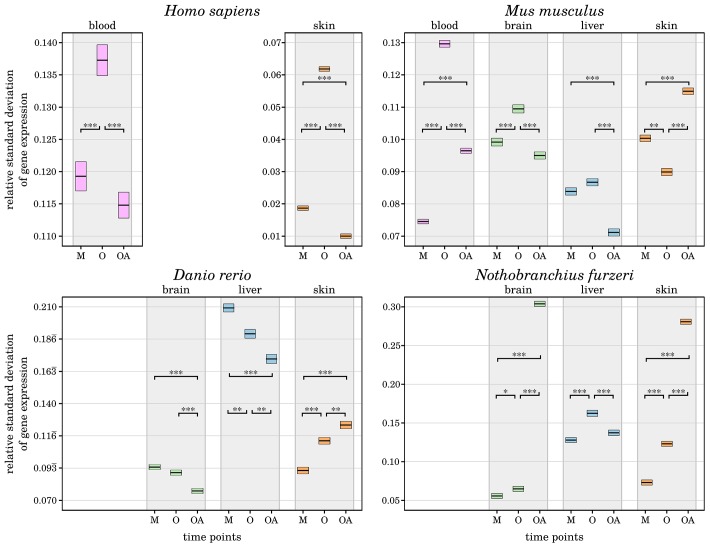
**Change of relative standard deviations of gene expression in all four species with age.** For each investigated species and tissue, the measured variance in transcript expression of the preselected senescence- and inflammation-related genes is displayed for every time point (M – mature, O – old, OA – old-aged). The upper and lower bounds of the box plots represent the respective 2.5% percentiles. All displayed differences in the mean variance of gene expression among the aged and old-aged time points and almost all other age comparisons are significant (*: p-Value ≤ 0*.*01, **: p-Value ≤ 0*.*001, ***: p-Value ≤ 0*.*0001,) within each tissue and species, individually. A general decrease of variance in gene expression can be observed in the old-age time points, except for the skin samples of *Mus musculus*, *Danio rerio* and *Nothobranchius furzeri*. This indicates a tighter control of inflammaging processes in long-lived individuals, reducing negative effects and helped them to reach the high age. For detailed information, see Supplementary Data 4.

It is proposed that individuals that have reached a comparatively high age have somehow counteracted the effects of inflammaging by unknown anti-inflammation processes [5, 6]. As one approach to validate this hypothesis, we analyzed how the mean variance in gene expression changes during aging. Interestingly, we found a significantly lower relative standard deviation of the old-age time points compared with the aged time points in almost all species and tissues and even lower than the mature time points in selected cases (see Figure 2 and Supplementary Data 4). This observation indicates that the selected genes associated with inflammation and senescence have a more stable expression, suggesting that both age-driving processes are in fact more controlled in the long-lived individuals. Similar observations were recently made in brains of long-lived mice [10]. Exceptions are the skin samples of all species other than humans, which show the opposite pattern of a significantly increased variance in gene expression in the old-age time point. The skin is the most diverse of the tissues studied in this work, with mice and humans having fur and hair, whereas fish have scales. In addition, it is one of the most exposed tissues and therefore faces a potentially higher diversity of factors that affect ageing in skin [11]. As a result, skin cells are subjected to more and different stresses, and the rate of senescence is increased compared with other tissues, which also affects the rate of inflammation within the skin tissues due to the pro-inflammatory secretory phenotype that senescent cells can develop [12]. In contrast, human skin showed a decreased variance in gene expression, indicating less pronounced or at least tighter controlled inflammatory and senescence processes. Such a distinct behavior could be the result of various intrinsic or extrinsic physiological factors like the thinning of the epidermis, the degeneration of the extra cellular matrix, or a decline of the microvasculature due to a reduced angiogenic capacity [24]. Besides, additional factors like external applications of antioxidants on skin might also play role in it [11].

The only other observed exception is the brain of *Nothobranchius furzeri*, which displayed a strongly increased gene expression variance at the old-age time point compared with both younger time points. This pattern is similar to that in fish skin and is already reflected in the sample clustering as mentioned above (see Figure 1). This observation could indicate a weaker protection of the *Nothobranchius furzeri* brain against inflammaging, but it remains to be further studied.

To verify that the more controlled gene expression observed in long-lived individuals is not biased due to our preselected genes, we repeated the same analysis with all expressed genes in every species and tissue. We confirmed our observation with all tissues showing the same relative variance changes as before, in principal. The overall variance within each age group was decreased, which was expected because many more genes were included in the analysis that show no change in expression during aging (see Supplementary Data 4). Nevertheless, in order to confirm that long-lived individuals really benefited from controlled gene expression, further investigations, such as longitudinal studies, are necessary.

### Oxidative stress response tends to be the prevailing process towards old age

**Figure 3:**
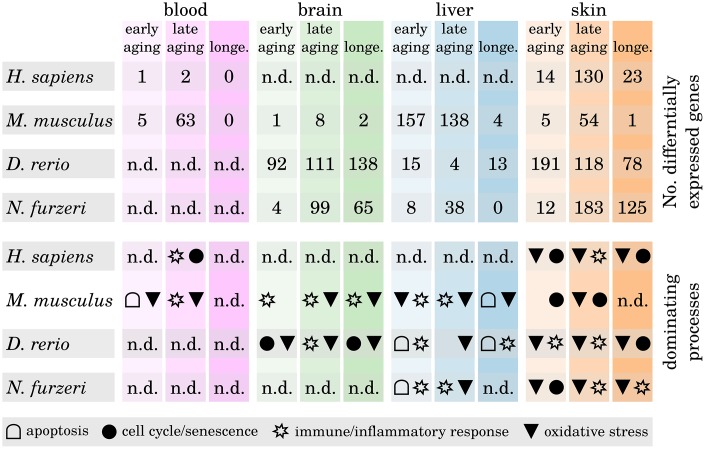
**Differentially expressed genes during tissue aging and associated biological processes.** The upper part shows the number of identified differentially expressed genes (DEGs) within all three age comparisons (early aging, late aging, longevity) in every investigated species and tissue. The lower part shows for every comparison the dominant biological processes as determined by the majority of the annotated functions of the respective DEGs. Note that in some comparisons only few DEGs could be identified and hence may only poorly reflect the underlying age-related processes (n.d. – no data). For detailed information, see Supplementary Data 6 and Supplementary Data 11.

To examine whether certain biological functions predominantly drive the inflammaging processes in a tissue- or species-dependent manner, we analyzed the identified differentially expressed genes (DEGs) with respect to their molecular function. We found that most of the DEGs within the brain and liver samples belong to immune and inflammatory response processes, whereas changes in the expression of senescence-related genes are predominately related to skin aging (see Figure 3, Supplementary Data 6 and Supplementary Data 11). Only few DEGs could be identified in blood age comparisons for humans and mice, making it difficult to interpret these results in a meaningful way.

It is a known and common observation of aged skin that it is more susceptible to infections, physical damage and reduced epidermal barrier integrity as well as other age-related deficiencies [14]. Epidermal stem cells maintain the tissue’s homeostasis and loss of those stem cells due to premature senescence is the main cause of aging within the skin [15]. Our data confirm this observation, showing mainly senescence-related DEGs within the skin of all four species already in the early and the late aging comparisons. However, whether the source of epidermal stem cell senescence is primarily intrinsic or extrinsic factors is controversially discussed [16, 17]. Apoptosis appears to play a more important role during aging of the liver than in any of the other investigated tissues. This is a confirmed observation, because liver homeostasis is mainly regulated through apoptotic processes and many liver dysfunctions and diseases are related to apoptosis [18, 19].

Most interestingly, we observed that DEGs associated with the oxidative stress response and oxidative processes occur predominantly within the late age and longevity comparisons (i.e., comparisons with the long-lived individuals) of the brain, liver and skin samples. This observation suggests that those individuals that grew older than their species average survival age have either a generally more active or better regulated response mechanism against reactive oxygen species (ROS). Free radicals such as ROS and their great impact on the aging process are much discussed [8, 20]. During respiration of oxygen, free radicals are produced as a harmful byproduct that can oxidize macromolecules such as DNA or proteins and damage them in this process. The main point of aging theories involving oxidative stress is that with age, the rate of harmful ROS accelerates because ROS first target the mitochondria (because they are mainly generated in that location), leading to an accumulation of damage in these cell organelles. The more degenerated the mitochondria become, the higher the rate of released ROS becomes, injuring genomes and membranes of neighboring cells and, as a final after-effect, resulting in their senescence or apoptosis and therefore aging [21]. Different antioxidant mechanisms exist within the cells to prevent the release of free radicals and protect the cells from such deleterious consequences [22]. It was observed that long-lived species appear to have higher protection against ROS than short-lived species [23, 24]. Our data suggest that the same is true for long-lived individuals within one species, confirming the importance of oxidative stress response for longevity.

### Conserved aging expression signatures across tissues and species

To identify a more precise signature of the abovementioned observations (Figure 3), we analyzed the expression patterns of our preselected gene set in greater detail. We were particularly interested in genes that show a constant increase or decrease towards old age that could also be consistently observed between the investigated tissues and species.

**Figure 4:**
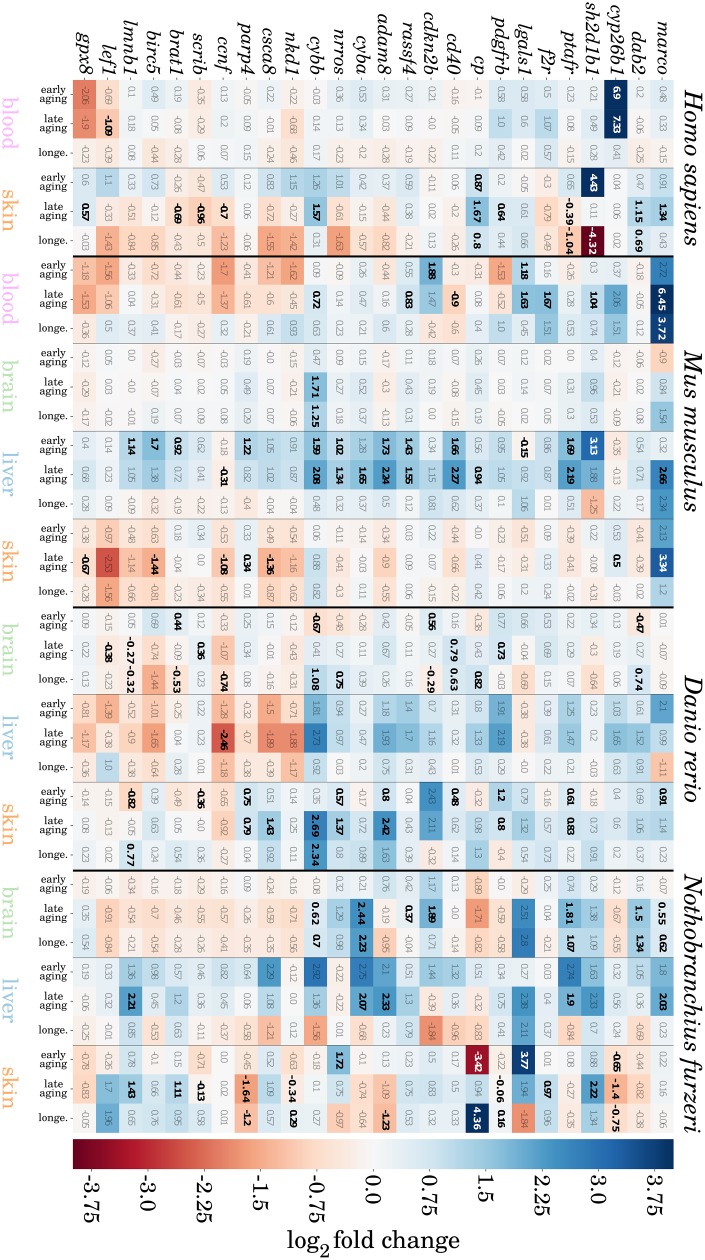
**Heatmap representation of potentially conserved senescence- and inflammation-related genes.** Genes are represented as mouse orthologues (a complete list of gene orthologues can be found in Supplementary Data 8). Numbers indicate *log*_2_ fold changes between two compared ages, where a positive value indicates an upregulation (blue), and a negative value downregulation (red) of the respective gene with aging. All significant changes in gene expression are indicated in bold. For detailed information, see Supplementary Data 7.

We found 16 genes that were consistently higher expressed and 10 that were consistently lower expressed during aging in at least three of the four species and tissues, totaling 26 genes that show a conserved expression pattern with aging (see Figure 4). Certain of these genes have already been reported in the context of aging, and we observed them to be the most conserved genes from our preselected set among species and tissues. Hence, we discuss several of these genes based on their biological function in additional detail. More senescence- and inflammation-related genes were found to have conserved expression patterns but to a lesser extent and can be found in Supplementary Data 7.

### Immune/inflammatory response

The gene *marco* encodes for a scavenger receptor (Marco – macrophage receptor with collagenous structure), which is typically found on macrophages but also other immune cells [25, 26], and acts in the innate immune system by binding and clearing pathogens and initiating an inflammatory response [27, 28]. We observed *marco* to be constantly upregulated (mostly by an increase of two-fold or more) towards old-age in all four species and within the blood, liver and skin. Although Marco does not cause inflammation directly, it is important for the activation of other receptors, such as members of the toll-like family [28]. These receptors subsequently activate NF-*κ*B, inducing an inflammatory response. Interestingly, Marco is associated with Alzheimer’s disease, because it is also present on microglia and a decreased response of these cells has been observed after binding amyloid beta peptides [29].

The coagulation factor II receptor (PAR1) is encoded by the gene *f2r*, which similar to *marco* shows persistent upregulation with age in blood, liver or skin within all four species. The major function of PAR1 is mediation between coagulation and inflammation activity, and therefore it has a key role in inflammatory response activation [30, 31]. In addition, PAR1 activity is implicated in aging-associated cardiovascular diseases [32].

Four more genes (*cd40*, *sh2d1b1*, *ptafr*, *adam8*) coding for membranous receptors have a common upregulated signature through several aging tissues and species. These genes have all been shown to promote pro-inflammatory signaling, either directly by binding platelet-activating factors (*ptafr* [33]), activating antigen presenting cells (*cd40* [34]), and acting as a regulator of antigen receptor signal transduction (*sh2d1b1* [35]) or more indirectly by releasing and degrading other cell surface receptors of leukocytes (*adam8* [36]).

Observation of a generally higher expression of ceruloplasmin encoded by the gene *cp* in all species and tissues with age (except *Nothobranchius furzeri* brain) is not unexpected due to its role as an acute-phase protein. These reactants are increasingly released in the blood plasma during an inflammatory response and support the innate immune response [37]. Thus, increased *cp* expression is most likely a result of the chronic inflammatory nature of aged tissues.

*Lef1* is one of the few immune-related genes that showed a decreased expression with age in the blood and skin of human and mice respectively and the brains of both examined fish. The main function of the LEF1 (lymphoid enhancer-binding factor 1) protein is to enhance expression of the T-cell receptor alpha chain [38]. However, a variety of different interaction partners are known, linking LEF1 as an important regulator within the WNT and TGF pathways and implicating a role in apoptosis and cell proliferation of leukocytes [39, 40].

### Oxidative stress response

As previously mentioned, ROS and oxidative stress are widely believed to be the driving force behind the cellular aging process [8]. The source of oxidative stress is likely an imbalanced activity of the respiratory chain and antioxidant processes. We observed a constant age-depended upregulation of the genes *cyba*, *cybb*, *cyp26b1* and *nrros* in various combinations of the investigated tissues and species. The first two genes encode for the light and heavy chains of the Cytochrome b-245 protein, a superoxide-producing subunit of the NADPH oxidase [41]. By producing and releasing ROS, phagocytes use Cytochrome b-245, mainly as an antimicrobial strategy during an infection. However, the role of Cytochrome b-245 during aging is still debated [42, 43], and it is associated with several degenerative diseases due to uncontrolled production of ROS [44, 45]. *Cyp26b1* encodes another cytochrome protein, Cytochrome P450, which is involved in many metabolic reactions, especially the oxidation of NADPH [46]. Higher expression of *cyp26b1* during aged time points is suggested to aid in degradation of toxic substances that have accumulated with age [47]. The genes *nrros* and *gpx8* both code for proteins that belong to antioxidant mechanisms controlling the production of ROS. NRROS directly interacts with Cytochrome b-245 and mediates its degradation, limiting the rate of ROS production of the associated NADPH oxidase complex [48]. Similarly, the enzyme GPX8 protects cells from ROS-induced damage by its peroxidase activity by reducing free hydrogen peroxide to water [49]. However, whereas gene expression of *nrros* rises with age, most likely to regulate the increased Cytochrome b-245 activity, the translation of *gpx8* is downregulated in many tissues and species. We observed the opposite regulation during aging only in the human skin and mouse liver samples. Nevertheless, *gpx8* serves as an interesting potential therapeutic target and it was previously shown in the *Caenorhabditis elegans* model that the deletion of several GPx family members is the cause of an accelerated aging process that results in shorter life-span [50].

### Senescence and apoptosis

Most of the 26 identified genes with conserved expression patterns in aging, are strongly related to the cell cycle control or apoptotic processes. Both replicative senescence and apoptosis are immediate causes for the loss of somatic and stem cells with age and can be similarly triggered by chronic inflammation and oxidative stress [51, 52].

We found four of these genes to encode direct inducers of senescence: *ccnf* (Cyclin-F) [53], *scrib* [54]. *cdkn2b* (p15^*INK*4*b*^) [55] and *rassf4* encoding an inhibitor of Cyclin D1 [56]. We found all of these genes to be expressed in a senescence-promoting fashion towards aging (*rassf4* and *cdkn2b* upregulated; *scrib* and *ccnf* downregulated) in the examined tissues and species. Additionally, selected genes interacting with cell cycle regulators also showed persistent expression towards old age, namely, *dab2* [57], *lgals1* [58], and *brat1* [59]. The proteins CDCA8 and Survivin encoded by *cdca8* and *birc5*, respectively, are both associated with apoptosis. By inhibiting caspase activity, Survivin is a repressor of apoptosis, whereas CDCA8 directly interacts and stabilizes Survivin [60, 61]. However, both proteins show converse directions of regulation during aging between the same tissues and within individual species, resembling rather weakly conserved expression signatures.

### Potential common tissue-specific marker genes of aging

**Figure 5:**
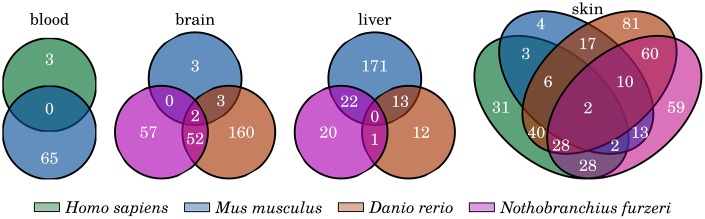
**Common inflammation- and senescence-related genes that are significantly changed with age.** Venn diagrams showing the overlap of the identified differentially expressed genes among the four investigated tissues. Only few genes are commonly differentially expressed among all species of any of the four tissue comparisons. For detailed information, see Supplementary Data 9.

We also focused on senescence- and inflammation-related genes that change their expression during aging consistently and similarly in a tissue-specific manner. These genes should present conserved age-related changes and might be the driving factors of tissue-specific aspects of the inflammaging process, probably closely linked to the specialized function of the respective tissue. In a first comparison of the identified DEGs of the different species, we observed only a few common genes that occur as differentially expressed in all species of a single tissue (see Figure 5 and Supplementary Data 9). However, the blood and liver samples revealed no DEGs to be common in all of the respective species, and two were identified within the brain (*cybb*, *cd68*) and another two within the skin (*Gpx7*, *Tnfrsf25*). *Cd68* is a member of the cell surface receptors of macrophages and other monocytes and is a well-known marker for macrophage activation. The Cytochrome b-245 subunit encoding gene *cybb* and its potentially harmful role during aging were already introduced in the previous section. Tnfrsf25 (a member of the TNF receptor superfamily) stimulates the proliferation of T cells or can initiate apoptotic signaling, leading either to survival or cell death, and is therefore an important regulator of T cell development [62]. *Gpx7* encodes for another glutathione peroxidase and has the same antioxidant function as its above described homolog *gpx8*.

However, none of the four genes exhibits features that serve as a tissue-specific marker because they do not display a consistent up- or downregulation during aging within each investigated species or share the same expression pattern with other tissues. Therefore, we applied a similar but more stringent filtering process to reveal genes displaying the characteristics of potential tissue-specific markers. With this approach, we identified several genes that showed a constant age-related transcriptional increase exclusively in the liver or skin samples of the investigated species.

### Liver

Four constantly upregulated genes were identified in the liver samples of *Mus musculus*, *Danio rerio* and *Nothobranchius furzeri*: *jag2*, *anax1*, *ralb*, and *sfrb*. The protein encoded by *jag2* (Jagged-2) is a known activating ligand of Notch2, which is mainly involved in many different developmental processes regulating cell fate decisions [63]. Most interestingly, *jag2* overexpression is not only shown to play a critical role in the formation of plasma cell myeloma and progression of other tumors [64, 65] but also induces the secretion of interleukin-6 [66]. Interleukin-6 is a potent stimulator of immune and inflammatory responses and acts in the development and progression of many age-associated diseases, such as Alzheimer’s disease [67], atherosclerosis [68], diabetes [69] and various cancers, making it an attractive therapeutic target [70, 71]. Because the liver shows strong signs of inflammation during aging (Figure 3), overexpression of *jag2* might be one conserved driving factor of inflammatory processes within hepatic cells and could be of interest as an additional therapeutic target. In contrast, Annexin A1, the protein encoded by *anax1*, has distinct anti-inflammatory and protective properties, by inhibiting NF-*κ*B signal transduction and counterregulating pro-inflammatory signals in a variety of immune cells [72, 73]. The age-related upregulation of Annexin A1 in the liver could be a component of a tissue-specific mechanism attempting to cope with the chronic state of inflammation. SFRP2 (encoded by *sfrp2*) has an oncogenic character and is associated with cancer formation by acting as an inhibitor of the canonical WNT/*β*catenin pathway [74, 75]. The last of the four conserved liver-specific upregulated genes, *ralb*, encodes for one of two Ral protein paralogs (Ral-B) and acts as a major modulator of a multitude of cellular processes [76]. Although many functions are shared between the paralogs Ral-A and Ral-B, the latter is more specifically involved in the activation of apoptosis [77], which we observed to occur more predominantly during liver aging (Figure 3). Additionally, Ral-B has clinical significance because it promotes tumor progression of several cancers and activation of the innate immune response [76, 78] and hence might contribute to inflammatory stress in the liver.

### Skin

If including all four species, we did not observe any gene displaying the characteristics of a potential conserved marker gene for skin aging. Considering the diverse nature of skin among the studied species we decided to examine the skin in two separate groups of, fishes and mammals.

Between human and mouse, only *pecam1* was identified to be consistently upregulated towards old age. The gene encodes the protein Pecam-1, also known as CD31, which is a common immunohistochemistry marker used to evaluate tumor growth and has a major role in the removal of old neutrophils, which could explain its higher expression with age. However, this gene is already known as a biomarker of inflammatory processes [79].

The two fishes *Danio rerio* and *Nothobranchius furzeri* share 6 genes that showed a persistent increased expression with aging exclusively in their skin and their encoded proteins are all involved in mitotic progression of the cell cycle: *ckap2* [80], *cdca8* [61], *aurkc* [81], *cep55* [82], *mis12* [83] and *spc25* [84]. This observation might indicate that fish skin cells are not as affected by senescence as the skin of humans and mice, which is in line with the observation that the skin of zebrafish shows a high regenerative capacity even at higher age [85].

## CONCLUSIONS

In the current descriptive study, we have investigated the inflammation- and senescence-related gene expression during aging in multiple tissues of four evolutionarily distinct species. By analyzing the gene expression profiles, we were able to identify common signatures of aging, *i.e.*, genes that illustrate steady increased or decreased expression with age. We identified 26 genes that shared an age-dependent expression pattern in at least three of the four investigated species and at least three different tissues. These genes represent interesting targets for further study, because they hint at general molecular mechanisms of aging that occur similarly not only within various organs of one organism but also between different species. It is important to note that we focused our study on a specific set of senescence- and inflammation-related genes and other processes might exist that share similar expression changes during aging across distinct species.

Several of the identified genes are not only directly involved in the initiation of an inflammatory response but also play a major role in sustaining the state of inflammation. Modulation of the expression level of certain these genes, such as *marco* and *f2r*, or addressing the respective proteins could be one potential approach to controlling chronic inflammation and thus might result in reduced inflammaging-related cellular stress with aging. In total, six different genes coding for macrophage cell surface receptors (*marco*, *f2r*, *cd40*, *sh2d1b1*, *ptafr*, *adam8*) have been found to show conserved upregulated expression across different tissues and species. Accumulation and higher activity of macrophages has already been linked to aging and removal of macrophages has shown beneficial effects for age-related disorders, such as neuro-degeneration, or atherosclerosis [86–88].

Another strongly aging-associated intrinsic stress factor is the production of cytotoxic ROS as a byproduct of the respiratory chain. Although phagocytes use Cytochrome b-245 (a heterodimer encoded by the genes *cyba* and *cybb*) to produce ROS to kill microbes, excessive generation of ROS causes premature replicative senescence of cells due to DNA damage [89]. We observed a conserved age-dependent upregulation of selected genes involved in ROS production (*cyba*, *cybb*, *cyp26b1*) together with a similarly upregulated but apparently inefficient (*nrros*) or even downregulated oxidative stress response (*gpx8*). Most of the identified genes with conserved expression patterns were related to senescence and apoptosis and showed an increase in both processes with age in general. As noted by many different theories on aging, each of the mentioned cellular processes is suspected to be the main driving force behind biological aging. However, it has become increasingly apparent that these processes should not be treated as separate and seemingly competing sources of aging, because they are strongly interconnected [89]. During a state of inflammatory stress, excessive production of ROS is enhanced due to dysfunction of the respiratory chain in mitochondria, leading to an accumulation of senescent cells, which can develop a pro-inflammatory secretory phenotype [12]. In addition, inflammation is a known trigger of apoptotic processes and during the process of apoptosis, certain cellular components are released that can further activate inflammatory processes, adding to the overall cellular stress [90]. As a consequence, an environment of chronic inflammatory, oxidative, senescent and apoptotic stress is established, with these processes mutually triggering each other and boosting the cycle of self-harming development [90] over time. Additionally, constant stimulation of cellular stress response mechanisms promotes genetic deregulation, which is reflected in the observed gene expression changes.

Based on the annotated functions of the identified DEGs, we observed that the individual tissues express different aging-related processes more strongly than others. Although the impact on age-related expression changes of immune and inflammatory response processes was stronger in the blood, brain and liver samples of all investigated species, the skin samples displayed greater modulation of cell cycle and senescence-associated genes. This observation is most likely due to the specialized functions of these different organs. Only few tissue-specific and conserved regulated genes could be observed in the liver and skin and none in the blood and brain samples. Nevertheless, the identified genes potentially represent molecular sources for why certain aging-related processes appear more tissue-specific than others.

Additionally, we report that gene transcription in long-lived individuals is generally more controlled compared with average-lived individuals, showing significantly lower variance in gene expression in all tissues and species, except the skin. Maintaining a more stable transcriptional activity, not only for senescence and inflammatory response processes, appears to have a significant life-prolonging effect [91]. It is also considered that oxidative stress response processes were observed to be more regulated in the old-age comparisons, and a possible reason could be better management of oxidative processes and more efficient antioxidant mechanisms. However, this conclusion remains speculative until further experimental studies can prove this observation.

Overall, in our work, we describe previously unknown conserved transcriptional changes across different species and tissues as well as tissue-specific changes with age, supplying a complementary overview of how changes in gene expression relate to processes of aging. In addition, our findings could serve as a basis for new strategies in the development of therapies against aging-related diseases.

## MATERIALS AND METHODS

### High-throughput transcriptomic data acquisition

All RNA-Seq libraries involved in this study originate from the JenAge consortium (http://www.jenage.de/) and were first published by Irizar et al [1]. These libraries are accessible at NCBI’s Gene Expression Omnibus (*Homo sapiens*: GSE75337, GSE103232; *Mus musculus*: GSE75192, GSE78130; *Danio rerio*: GSE74244 and *Nothobranchius furzeri*: GSE52462, GSE66712).

### High-throughput transcriptomic data processing

**Figure 6:**
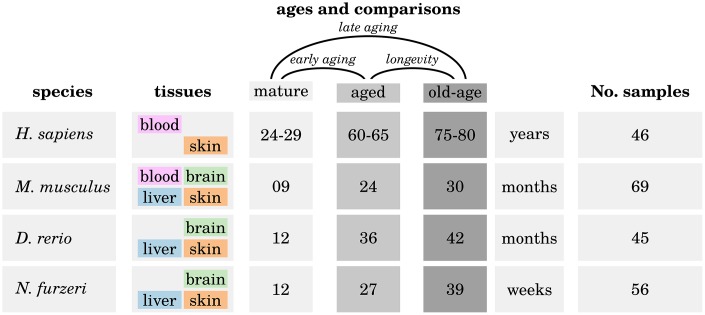
**Overview of the analyzed high-throughput transcriptomic data.** For each of the four investigated species, up to four different tissues were sampled at three different ages: one mature (M) time point, one aged (A) time point and one old-aged (OA) time point. Three different comparisons were made to reveal significant differences in expression of genes during early aging (M vs. A), late aging (M vs. OA) and longevity (O vs. OA). For more details, see Supplementary Data 1.

For each species we investigated up to four different tissues at three different ages: young mature (M), aged (A) and old-aged (OA) (see Figure 6).

First, *Prinseq* (v0.20.3) [92] was used to trim and filter the RNA-Seq libraries, by clipping reads on either ends to achieve a minimum base quality score of 20 and discarding all reads with a length of less than 15 nt or containing more than two ambiguous N bases. Second, quality-trimmed RNA-Seq libraries were mapped onto the according recent genomes (as from Ensembl release version 92 [93] and [94, 9]) using *TopHat2* (v2.1.1) [95] under consideration of the default parameters. This procedure allowed the identification of spliced reads, and the mapping of single reads to multiple best-fit locations. Third, read counting was performed by *Featurecounts* (v1.5.3) [96], with the counts normalized to transcripts per million (TPM) [97] to discard all genes with a value of ≤ 1 in every sample from subsequent expression analyses. Finally, differentially expressed genes were identified via the *DESeq2* (v1.10.0) [98] Bioconductor package by comparing the three different time points of each species and tissue. False discovery rate adjustment of the resulting gene’s p-values was performed according to previous work [99]. Details of all DEG results, together with the raw and normalized count values are given in detail in Supplementary Data 1.

### Collection of genes of interest and t-SNE dimensionality reduction

An initial list of 769 genes relevant in the context of senescence and inflammaging was compiled from a detailed literature search (see Supplementary Data 2). From those 769 genes, we removed all genes that were either missing an ortholog in any of the four species or were not differentially expressed (FDR adjusted p-value < 0.05) at least once in any species and tissue, ending with the final list of 464 genes (see Supplementary Data 3). Based on the measured expression of these 464 genes the t-SNE algorithm [100] was used for dimensionality reduction in order to investigate the clustering behavior of the analyzed RNA-Seq libraries (see Figure 1).

### Gene expression coefficient of variation analysis

To estimate the general gene expression variation among the different time points of each species and tissue, we calculated the coefficient of variation for every expressed gene (*i.e.*, a TPM value greater than 1) as the ratio of its standard deviation to its mean. The significance of the variation differences among time points was determined using the two-sided t-test. We repeated this analysis using only the 464 genes of interest (for details, Supplementary Data 4).

### Gene function annotation and expression pattern analysis

The exact biological functions of our preselected gene set of interest were obtained from the functional annotation database *David* (version 6.8) [101] (Supplementary Data 5). For every age comparison (Figure 6) we determined the dominant biological processes by the majority of the annotated functions of the respective DEGs (for details, see Supplementary Data 11). To identify common signatures of senescence- or inflammation-related processes, we filtered for genes that were differentially expressed at least once within one of the age comparisons and showed the same direction of up- or downregulation by a minimum threshold of 10% in every other age comparison. This rather low effect-size threshold can be justified, because it was previously shown that expression changes related to aging tend to be more subtle [102]. This process was conducted for every species and tissue individually. Based on this gene subset, we further searched for common expression patterns of single genes among the different tissues and species. When searching for tissue-specific signatures, we applied the same filtering strategy but raised the minimum threshold of expression changes to 25%.

### Additional information

All relevant data can be found within the paper and its supporting information files. The online supplement is integrated into the open science framework (https://osf.io/) and available under https://osf.io/kzq5y/ or https://doi.org/10.17605/OSF.IO/KZQ5Y. As soon as this manuscript gets published, the Supplementary Data will be made publicly available and the corresponding link and doi will be provided

## References

[1] Aramillo Irizar P, Schäuble S, Esser D, Groth M, Frahm C, Priebe S, Baumgart M, Hartmann N, Marthandan S, Menzel U, Müller J, Schmidt S, Ast V, et al. Transcriptomic alterations during ageing reflect the shift from cancer to degenerative diseases in the elderly. Nat Commun. 2018; 9:327. 10.1038/s41467-017-02395-2 29382830 PMC5790807

[2] Collado M, Blasco MA, Serrano M. Cellular senescence in cancer and aging. Cell. 2007; 130:223–33. 10.1016/j.cell.2007.07.003 17662938

[3] Jeyapalan JC, Sedivy JM. Cellular senescence and organismal aging. Mech Ageing Dev. 2008; 129:467–74. 10.1016/j.mad.2008.04.001 18502472 PMC3297662

[4] Bhatia-Dey N, Kanherkar RR, Stair SE, Makarev EO, Csoka AB. Cellular senescence as the causal nexus of aging. Front Genet. 2016; 7:13. 10.3389/fgene.2016.00013 26904101 PMC4751276

[5] Giunta B, Fernandez F, Nikolic WV, Obregon D, Rrapo E, Town T, Tan J. Inflammaging as a prodrome to Alzheimer’s disease. J Neuroinflammation. 2008; 5:51. 10.1186/1742-2094-5-51 19014446 PMC2615427

[6] Vasto S, Caruso C. Immunity &Ageing: a new journal looking at ageing from an immunological point of view. Immun Ageing. 2004; 1:1. 10.1186/1742-4933-1-1 15679921 PMC544954

[7] Franceschi C, Capri M, Monti D, Giunta S, Olivieri F, Sevini F, Panourgia MP, Invidia L, Celani L, Scurti M, Cevenini E, Castellani GC, Salvioli S. Inflammaging and anti-inflammaging: a systemic perspective on aging and longevity emerged from studies in humans. Mech Ageing Dev. 2007; 128:92–105. 10.1016/j.mad.2006.11.016 17116321

[8] De la Fuente M, Miquel J. An update of the oxidation-inflammation theory of aging: the involvement of the immune system in oxi-inflamm-aging. Curr Pharm Des. 2009; 15:3003–26. 10.2174/138161209789058110 19754376

[9] Baumgart M, Barth E, Savino A, Groth M, Koch P, Petzold A, Arisi I, Platzer M, Marz M, Cellerino A. A miRNA catalogue and ncRNA annotation of the short-living fish Nothobranchius furzeri. BMC Genomics. 2017; 18:693. 10.1186/s12864-017-3951-8 28874118 PMC5584509

[10] Frahm C, Srivastava A, Schmidt S, Mueller J, Groth M, Guenther M, Ji Y, Priebe S, Platzer M, Witte OW. Transcriptional profiling reveals protective mechanisms in brains of long-lived mice. Neurobiol Aging. 2017; 52:23–31. 10.1016/j.neurobiolaging.2016.12.016 28110102

[11] Zhang S, Duan E. Fighting against skin aging: the way from bench to bedside. Cell Transplant. 2018; 27:729–38. 10.1177/0963689717725755 29692196 PMC6047276

[12] Campisi J. Aging, cellular senescence, and cancer. Annu Rev Physiol. 2013; 75:685–705. 10.1146/annurev-physiol-030212-183653 23140366 PMC4166529

[13] Scioli MG, Bielli A, Arcuri G, Ferlosio A, Orlandi A. Ageing and microvasculature. Vasc Cell. 2014; 6:19. 10.1186/2045-824X-6-19 25243060 PMC4169693

[14] McCullough JL, Kelly KM. Prevention and treatment of skin aging. Ann N Y Acad Sci. 2006; 1067:323–31. 10.1196/annals.1354.044 16804006

[15] Ressler S, Bartkova J, Niederegger H, Bartek J, Scharffetter-Kochanek K, Jansen-Dürr P, Wlaschek M. p16INK4A is a robust in vivo biomarker of cellular aging in human skin. Aging Cell. 2006; 5:379–89. 10.1111/j.1474-9726.2006.00231.x 16911562

[16] Stern MM, Bickenbach JR. Epidermal stem cells are resistant to cellular aging. Aging Cell. 2007; 6:439–52. 10.1111/j.1474-9726.2007.00318.x 17635170

[17] Giangreco A, Qin M, Pintar JE, Watt FM. Epidermal stem cells are retained in vivo throughout skin aging. Aging Cell. 2008; 7:250–59. 10.1111/j.1474-9726.2008.00372.x 18221414 PMC2339763

[18] Guicciardi ME, Gores GJ. Apoptosis: a mechanism of acute and chronic liver injury. Gut. 2005; 54:1024–33. 10.1136/gut.2004.053850 15951554 PMC1774601

[19] Guicciardi ME, Malhi H, Mott JL, Gores GJ. Apoptosis and necrosis in the liver. Compr Physiol. 2013; 3:977–1010. 10.1002/cphy.c120020 23720337 PMC3867948

[20] Miquel J. An update on the oxygen stress-mitochondrial mutation theory of aging: genetic and evolutionary implications. Exp Gerontol. 1998; 33:113–26. 10.1016/S0531-5565(97)00060-0 9467721

[21] Miquel J, Fleming J. Theoretical and experimental support for an “oxygen radical-mitochondrial injury” hypothesis of cell aging. Free radicals, aging degenerative diseases. New York, 1986; 51–74.

[22] Sies H. Biochemistry of oxidative stress. Angew Chem Int Ed Engl. 1986; 25:1058–71. 10.1002/anie.198610581

[23] Barja G. Free radicals and aging. Trends Neurosci. 2004; 27:595–600. 10.1016/j.tins.2004.07.005 15374670

[24] Pamplona R, Barja G. Aging rate, mitochondrial free radical production, and constitutive sensitivity to lipid peroxidation: insights from comparative studies. Aging at the molecular level. Springer; 2003 pp. 47–64. 10.1007/978-94-017-0667-4_4

[25] Plüddemann A, Neyen C, Gordon S. Macrophage scavenger receptors and host-derived ligands. Methods. 2007; 43:207–17. 10.1016/j.ymeth.2007.06.004 17920517

[26] Bowdish DM, Gordon S. Conserved domains of the class A scavenger receptors: evolution and function. Immunol Rev. 2009; 227:19–31. 10.1111/j.1600-065X.2008.00728.x 19120472

[27] Mukhopadhyay S, Varin A, Chen Y, Liu B, Tryggvason K, Gordon S. SR-A/MARCO-mediated ligand delivery enhances intracellular TLR and NLR function, but ligand scavenging from cell surface limits TLR4 response to pathogens. Blood. 2011; 117:1319–28. 10.1182/blood-2010-03-276733 21098741

[28] Novakowski KE, Huynh A, Han S, Dorrington MG, Yin C, Tu Z, Pelka P, Whyte P, Guarné A, Sakamoto K, Bowdish DM. A naturally occurring transcript variant of MARCO reveals the SRCR domain is critical for function. Immunol Cell Biol. 2016; 94:646–55. 10.1038/icb.2016.20 26888252 PMC4980223

[29] Yu Y, Ye RD. Microglial Aβ receptors in Alzheimer’s disease. Cell Mol Neurobiol. 2015; 35:71–83. 10.1007/s10571-014-0101-6 25149075 PMC11486233

[30] Coughlin SR, Vu TK, Hung DT, Wheaton VI. Characterization of a functional thrombin receptor. Issues and opportunities. J Clin Invest. 1992; 89:351–5. 10.1172/JCI115592 1310691 PMC442859

[31] Wu H, Zhang Z, Li Y, Zhao R, Li H, Song Y, Qi J, Wang J. Time course of upregulation of inflammatory mediators in the hemorrhagic brain in rats: correlation with brain edema. Neurochem Int. 2010; 57:248–53. 10.1016/j.neuint.2010.06.002 20541575 PMC2910823

[32] Leger AJ, Covic L, Kuliopulos A. Protease-activated receptors in cardiovascular diseases. Circulation. 2006; 114:1070–77. 10.1161/CIRCULATIONAHA.105.574830 16952995

[33] Honda Z, Ishii S, Shimizu T. Platelet-activating factor receptor. J Biochem. 2002; 131:773–79. 10.1093/oxfordjournals.jbchem.a003164 12038971

[34] Chatzigeorgiou A, Lyberi M, Chatzilymperis G, Nezos A, Kamper E. CD40/CD40L signaling and its implication in health and disease. Biofactors. 2009; 35:474–83. 10.1002/biof.62 19904719

[35] Morra M, Lu J, Poy F, Martin M, Sayos J, Calpe S, Gullo C, Howie D, Rietdijk S, Thompson A, Coyle AJ, Denny C, Yaffe MB, et al. Structural basis for the interaction of the free SH2 domain EAT-2 with SLAM receptors in hematopoietic cells. EMBO J. 2001; 20:5840–52. 10.1093/emboj/20.21.5840 11689425 PMC125701

[36] Yamamoto S, Higuchi Y, Yoshiyama K, Shimizu E, Kataoka M, Hijiya N, Matsuura K. ADAM family proteins in the immune system. Immunol Today. 1999; 20:278–84. 10.1016/S0167-5699(99)01464-4 10354553

[37] Hellman NE, Gitlin JD. Ceruloplasmin metabolism and function. Annu Rev Nutr. 2002; 22:439–58. 10.1146/annurev.nutr.22.012502.114457 12055353

[38] Milatovich A, Travis A, Grosschedl R, Francke U. Gene for lymphoid enhancer-binding factor 1 (LEF1) mapped to human chromosome 4 (q23-q25) and mouse chromosome 3 near Egf. Genomics. 1991; 11:1040–48. 10.1016/0888-7543(91)90030-I 1783375

[39] Gandhirajan RK, Staib PA, Minke K, Gehrke I, Plickert G, Schlösser A, Schmitt EK, Hallek M, Kreuzer KA. Small molecule inhibitors of Wnt/β-catenin/lef-1 signaling induces apoptosis in chronic lymphocytic leukemia cells in vitro and in vivo. Neoplasia. 2010; 12:326–35. 10.1593/neo.91972 20360943 PMC2847740

[40] Labbé E, Letamendia A, Attisano L. Association of Smads with lymphoid enhancer binding factor 1/T cell-specific factor mediates cooperative signaling by the transforming growth factor-β and wnt pathways. Proc Natl Acad Sci USA. 2000; 97:8358–63. 10.1073/pnas.150152697 10890911 PMC26952

[41] Hervé C, Tonon T, Collén J, Corre E, Boyen C. NADPH oxidases in Eukaryotes: red algae provide new hints! Curr Genet. 2006; 49:190–204. 10.1007/s00294-005-0044-z 16344959

[42] Sahoo S, Meijles DN, Pagano PJ. NADPH oxidases: key modulators in aging and age-related cardiovascular diseases? Clin Sci (Lond). 2016; 130:317–35. 10.1042/CS20150087 26814203 PMC4818578

[43] Baciou L, Masoud R, Souabni H, Serfaty X, Karimi G, Bizouarn T, Houée Levin C. Phagocyte NADPH oxidase, oxidative stress and lipids: Anti- or pro ageing? Mech Ageing Dev. 2018; 172:30–34. 10.1016/j.mad.2017.11.001 29103982

[44] Bedard K, Krause KH. The NOX family of ROS-generating NADPH oxidases: physiology and pathophysiology. Physiol Rev. 2007; 87:245–313. 10.1152/physrev.00044.2005 17237347

[45] Hernandes MS, D’Avila JC, Trevelin SC, Reis PA, Kinjo ER, Lopes LR, Castro-Faria-Neto HC, Cunha FQ, Britto LR, Bozza FA. The role of Nox2-derived ROS in the development of cognitive impairment after sepsis. J Neuroinflammation. 2014; 11:36. 10.1186/1742-2094-11-36 24571599 PMC3974031

[46] Harayama S, Kok M, Neidle EL. Functional and evolutionary relationships among diverse oxygenases. Annu Rev Microbiol. 1992; 46:565–601. 10.1146/annurev.mi.46.100192.003025 1444267

[47] Zahn JM, Sonu R, Vogel H, Crane E, Mazan-Mamczarz K, Rabkin R, Davis RW, Becker KG, Owen AB, Kim SK. Transcriptional profiling of aging in human muscle reveals a common aging signature. PLoS Genet. 2006; 2:e115. 10.1371/journal.pgen.0020115 16789832 PMC1513263

[48] Noubade R, Wong K, Ota N, Rutz S, Eidenschenk C, Valdez PA, Ding J, Peng I, Sebrell A, Caplazi P, DeVoss J, Soriano RH, Sai T, et al. NRROS negatively regulates reactive oxygen species during host defence and autoimmunity. Nature. 2014; 509:235–39. 10.1038/nature13152 24739962

[49] Toppo S, Vanin S, Bosello V, Tosatto SC. Evolutionary and structural insights into the multifaceted glutathione peroxidase (Gpx) superfamily. Antioxid Redox Signal. 2008; 10:1501–14. 10.1089/ars.2008.2057 18498225

[50] Sakamoto T, Maebayashi K, Nakagawa Y, Imai H. Deletion of the four phospholipid hydroperoxide glutathione peroxidase genes accelerates aging in Caenorhabditis elegans. Genes Cells. 2014; 19:778–92. 10.1111/gtc.12175 25200408

[51] Liu X, Kim CN, Yang J, Jemmerson R, Wang X. Induction of apoptotic program in cell-free extracts: requirement for dATP and cytochrome c. Cell. 1996; 86:147–57. 10.1016/S0092-8674(00)80085-9 8689682

[52] Böhm I, Schild H. Apoptosis: the complex scenario for a silent cell death. Mol Imaging Biol. 2003; 5:2–14. 10.1016/S1536-1632(03)00024-6 14499155

[53] Wang Z, Liu P, Inuzuka H, Wei W. Roles of F-box proteins in cancer. Nat Rev Cancer. 2014; 14:233–47. 10.1038/nrc3700 24658274 PMC4306233

[54] Dow LE, Kauffman JS, Caddy J, Zarbalis K, Peterson AS, Jane SM, Russell SM, Humbert PO. The tumour-suppressor Scribble dictates cell polarity during directed epithelial migration: regulation of Rho GTPase recruitment to the leading edge. Oncogene. 2007; 26:2272–82. 10.1038/sj.onc.1210016 17043654

[55] Hannon GJ, Beach D. p15INK4B is a potential effector of TGF-beta-induced cell cycle arrest. Nature. 1994; 371:257–61. 10.1038/371257a0 8078588

[56] Zhang M, Wang D, Zhu T, Yin R. Rassf4 overexpression inhibits the proliferation, invasion, emt, and wnt signaling pathway in osteosarcoma cells. Oncol Res. 2017; 25:83–91. 10.3727/096504016X14719078133447 28081736 PMC7840746

[57] He J, Xu J, Xu XX, Hall RA. Cell cycle-dependent phosphorylation of Disabled-2 by cdc2. Oncogene. 2003; 22:4524–30. 10.1038/sj.onc.1206767 12881709

[58] Gauthier L, Rossi B, Roux F, Termine E, Schiff C. Galectin-1 is a stromal cell ligand of the pre-B cell receptor (BCR) implicated in synapse formation between pre-B and stromal cells and in pre-BCR triggering. Proc Natl Acad Sci USA. 2002; 99:13014–19. 10.1073/pnas.202323999 12271131 PMC130578

[59] So EY, Ouchi T. BRAT1 deficiency causes increased glucose metabolism and mitochondrial malfunction. BMC Cancer. 2014; 14:548. 10.1186/1471-2407-14-548 25070371 PMC4129107

[60] Sah NK, Khan Z, Khan GJ, Bisen PS. Structural, functional and therapeutic biology of survivin. Cancer Lett. 2006; 244:164–71. 10.1016/j.canlet.2006.03.007 16621243

[61] Gassmann R, Carvalho A, Henzing AJ, Ruchaud S, Hudson DF, Honda R, Nigg EA, Gerloff DL, Earnshaw WC. Borealin: a novel chromosomal passenger required for stability of the bipolar mitotic spindle. J Cell Biol. 2004; 166:179–91. 10.1083/jcb.200404001 15249581 PMC2172304

[62] Borysenko CW, Furey WF, Blair HC. Comparative modeling of TNFRSF25 (DR3) predicts receptor destabilization by a mutation linked to rheumatoid arthritis. Biochem Biophys Res Commun. 2005; 328:794–99. 10.1016/j.bbrc.2005.01.017 15694416

[63] Shimizu K, Chiba S, Hosoya N, Kumano K, Saito T, Kurokawa M, Kanda Y, Hamada Y, Hirai H. Binding of Delta1, Jagged1, and Jagged2 to Notch2 rapidly induces cleavage, nuclear translocation, and hyperphosphorylation of Notch2. Mol Cell Biol. 2000; 20:6913–22. 10.1128/MCB.20.18.6913-6922.2000 10958687 PMC88767

[64] Pietras A, von Stedingk K, Lindgren D, Påhlman S, Axelson H. JAG2 induction in hypoxic tumor cells alters Notch signaling and enhances endothelial cell tube formation. Mol Cancer Res. 2011; 9:626–36. 10.1158/1541-7786.MCR-10-0508 21402725

[65] Chiron D, Maïga S, Descamps G, Moreau P, Le Gouill S, Marionneau S, Ouiller T, Moreaux J, Klein B, Bataille R, Amiot M, Pellat-Deceunynck C. Critical role of the NOTCH ligand JAG2 in self-renewal of myeloma cells. Blood Cells Mol Dis. 2012; 48:247–53. 10.1016/j.bcmd.2012.01.006 22341562

[66] Houde C, Li Y, Song L, Barton K, Zhang Q, Godwin J, Nand S, Toor A, Alkan S, Smadja NV, Avet-Loiseau H, Lima CS, Miele L, Coignet LJ. Overexpression of the NOTCH ligand JAG2 in malignant plasma cells from multiple myeloma patients and cell lines. Blood. 2004; 104:3697–704. 10.1182/blood-2003-12-4114 15292061

[67] Swardfager W, Lanctôt K, Rothenburg L, Wong A, Cappell J, Herrmann N. A meta-analysis of cytokines in Alzheimer’s disease. Biol Psychiatry. 2010; 68:930–41. 10.1016/j.biopsych.2010.06.012 20692646

[68] Dubinski A, Zdrojewicz Z. [The role of interleukin-6 in development and progression of atherosclerosis.] Pol Merkur Lekarski. 2007; 22:291–294. 17684929

[69] Kristiansen OP, Mandrup-Poulsen T. Interleukin-6 and diabetes: the good, the bad, or the indifferent? Diabetes. 2005 (Suppl 2); 54:S114–24. 10.2337/diabetes.54.suppl_2.S114 16306329

[70] Barton BE. Interleukin-6 and new strategies for the treatment of cancer, hyperproliferative diseases and paraneoplastic syndromes. Expert Opin Ther Targets. 2005; 9:737–52. 10.1517/14728222.9.4.737 16083340

[71] Smolen JS, Maini RN. Interleukin-6: a new therapeutic target. Arthritis Res Ther. 2006 (Suppl 2); 8:S5. 10.1186/ar1969 16899109 PMC3226077

[72] Perretti M, D’Acquisto F. Annexin A1 and glucocorticoids as effectors of the resolution of inflammation. Nat Rev Immunol. 2009; 9:62–70. 10.1038/nri2470 19104500

[73] Zhang Z, Huang L, Zhao W, Rigas B. Annexin 1 induced by anti-inflammatory drugs binds to NF-kappaB and inhibits its activation: anticancer effects in vitro and in vivo. Cancer Res. 2010; 70:2379–88. 10.1158/0008-5472.CAN-09-4204 20215502 PMC2953961

[74] Kim H, Yoo S, Zhou R, Xu A, Bernitz JM, Yuan Y, Gomes AM, Daniel MG, Su J, Demicco EG, Zhu J, Moore KA, Lee DF, et al. Oncogenic role of SFRP2 in p53-mutant osteosarcoma development via autocrine and paracrine mechanism. Proc Natl Acad Sci USA. 2018; 115:E11128–37. 10.1073/pnas.1814044115 30385632 PMC6255152

[75] Ren J, Jian F, Jiang H, Sun Y, Pan S, Gu C, Chen X, Wang W, Ning G, Bian L, Sun Q. Decreased expression of SFRP2 promotes development of the pituitary corticotroph adenoma by upregulating Wnt signaling. Int J Oncol. 2018; 52:1934–46. 10.3892/ijo.2018.4355 29620167 PMC5919716

[76] Simicek M, Lievens S, Laga M, Guzenko D, Aushev VN, Kalev P, Baietti MF, Strelkov SV, Gevaert K, Tavernier J, Sablina AA. The deubiquitylase USP33 discriminates between RALB functions in autophagy and innate immune response. Nat Cell Biol. 2013; 15:1220–30. 10.1038/ncb2847 24056301

[77] Simicek M, Lievens S, Laga M, Guzenko D, Aushev VN, Kalev P, Baietti MF, Strelkov SV, Gevaert K, Tavernier J, Sablina AA. The deubiquitylase USP33 discriminates between RALB functions in autophagy and innate immune response. Nat Cell Biol. 2013; 15:1220–30. 10.1038/ncb2847 24056301

[78] Kashatus DF. Ral GTPases in tumorigenesis: emerging from the shadows. Exp Cell Res. 2013; 319:2337–42. 10.1016/j.yexcr.2013.06.020 23830877 PMC4270277

[79] Villar J, Zhang H, Slutsky AS. Lung repair and regeneration in ards: role of pecam1 and wnt signaling. Chest. 2019; 155:587–594. 10.1016/j.chest.2018.10.022 30392791 PMC6435939

[80] Maouche-Chrétien L, Deleu N, Badoual C, Fraissignes P, Berger R, Gaulard P, Roméo PH, Leroy-Viard K. Identification of a novel cDNA, encoding a cytoskeletal associated protein, differentially expressed in diffuse large B cell lymphomas. Oncogene. 1998; 17:1245–51. 10.1038/sj.onc.1202048 9771967

[81] Slattery SD, Mancini MA, Brinkley BR, Hall RM. Aurora-C kinase supports mitotic progression in the absence of Aurora-B. Cell Cycle. 2009; 8:2984–94. 10.4161/cc.8.18.9591 19713763

[82] van der Horst A, Simmons J, Khanna KK. Cep55 stabilization is required for normal execution of cytokinesis. Cell Cycle. 2009; 8:3742–49. 10.4161/cc.8.22.10047 19855176

[83] Obuse C, Iwasaki O, Kiyomitsu T, Goshima G, Toyoda Y, Yanagida M. A conserved Mis12 centromere complex is linked to heterochromatic HP1 and outer kinetochore protein Zwint-1. Nat Cell Biol. 2004; 6:1135–41. 10.1038/ncb1187 15502821

[84] McCleland ML, Kallio MJ, Barrett-Wilt GA, Kestner CA, Shabanowitz J, Hunt DF, Gorbsky GJ, Stukenberg PT. The vertebrate Ndc80 complex contains Spc24 and Spc25 homologs, which are required to establish and maintain kinetochore-microtubule attachment. Curr Biol. 2004; 14:131–37. 10.1016/j.cub.2003.12.058 14738735

[85] Lund TC, Glass TJ, Tolar J, Blazar BR. Expression of telomerase and telomere length are unaffected by either age or limb regeneration in Danio rerio. PLoS One. 2009; 4:e7688. 10.1371/journal.pone.0007688 19893630 PMC2766636

[86] Prattichizzo F, Bonafè M, Olivieri F, Franceschi C. Senescence associated macrophages and “macroph-aging”: are they pieces of the same puzzle? Aging (Albany NY). 2016; 8:3159–60. 10.18632/aging.101133 27941213 PMC5270660

[87] Childs BG, Baker DJ, Wijshake T, Conover CA, Campisi J, van Deursen JM. Senescent intimal foam cells are deleterious at all stages of atherosclerosis. Science. 2016; 354:472–77. 10.1126/science.aaf6659 27789842 PMC5112585

[88] Spangenberg EE, Lee RJ, Najafi AR, Rice RA, Elmore MR, Blurton-Jones M, West BL, Green KN. Eliminating microglia in Alzheimer’s mice prevents neuronal loss without modulating amyloid-β pathology. Brain. 2016; 139:1265–81. 10.1093/brain/aww016 26921617 PMC5006229

[89] López-Otín C, Blasco MA, Partridge L, Serrano M, Kroemer G. The hallmarks of aging. Cell. 2013; 153:1194–217. 10.1016/j.cell.2013.05.039 23746838 PMC3836174

[90] Vandivier RW, Henson PM, Douglas IS. Burying the dead: the impact of failed apoptotic cell removal (efferocytosis) on chronic inflammatory lung disease. Chest. 2006; 129:1673–82. 10.1378/chest.129.6.1673 16778289

[91] Franceschi C, Bonafè M, Valensin S, Olivieri F, De Luca M, Ottaviani E, De Benedictis G. Inflamm-aging. An evolutionary perspective on immunosenescence. Ann N Y Acad Sci. 2000; 908:244–54. 10.1111/j.1749-6632.2000.tb06651.x 10911963

[92] Schmieder R, Edwards R. Quality control and preprocessing of metagenomic datasets. Bioinformatics. 2011; 27:863–64. 10.1093/bioinformatics/btr026 21278185 PMC3051327

[93] Zerbino DR, Achuthan P, Akanni W, Amode MR, Barrell D, Bhai J, Billis K, Cummins C, Gall A, Girón CG, Gil L, Gordon L, Haggerty L, et al. Ensembl 2018. Nucleic Acids Res. 2018; 46:D754–61. 10.1093/nar/gkx1098 29155950 PMC5753206

[94] Reichwald K, Petzold A, Koch P, Downie BR, Hartmann N, Pietsch S, Baumgart M, Chalopin D, Felder M, Bens M, Sahm A, Szafranski K, Taudien S, et al. Insights into sex chromosome evolution and aging from the genome of a short-lived fish. Cell. 2015; 163:1527–38. 10.1016/j.cell.2015.10.071 26638077

[95] Kim D, Pertea G, Trapnell C, Pimentel H, Kelley R, Salzberg SL. TopHat2: accurate alignment of transcriptomes in the presence of insertions, deletions and gene fusions. Genome Biol. 2013; 14:R36. 10.1186/gb-2013-14-4-r36 23618408 PMC4053844

[96] Liao Y, Smyth GK, Shi W. featureCounts: an efficient general purpose program for assigning sequence reads to genomic features. Bioinformatics. 2014; 30:923–30. 10.1093/bioinformatics/btt656 24227677

[97] Wagner GP, Kin K, Lynch VJ. Measurement of mRNA abundance using RNA-seq data: RPKM measure is inconsistent among samples. Theory Biosci. 2012; 131:281–85. 10.1007/s12064-012-0162-3 22872506

[98] Love MI, Huber W, Anders S. Moderated estimation of fold change and dispersion for RNA-seq data with DESeq2. Genome Biol. 2014; 15:550. 10.1186/s13059-014-0550-8 25516281 PMC4302049

[99] Benjamini Y, Hochberg Y. Controlling the false discovery rate: a practical and powerful approach to multiple testing. J R Stat Soc Ser B. 1995; 57:289–300. 10.1111/j.2517-6161.1995.tb02031.x

[100] van der Maaten L, Hinton G. Visualizing data using t-sne. J Mach Learn Res. 2008; 9:2579–605.

[101] Huang da W, Sherman BT, Lempicki RA. Systematic and integrative analysis of large gene lists using DAVID bioinformatics resources. Nat Protoc. 2009; 4:44–57. 10.1038/nprot.2008.211 19131956

[102] de Magalhães JP, Finch CE, Janssens G. Next-generation sequencing in aging research: emerging applications, problems, pitfalls and possible solutions. Ageing Res Rev. 2010; 9:315–23. 10.1016/j.arr.2009.10.006 19900591 PMC2878865

